# Atmospheric Deposition Impact on Bacterial Community Composition in the NW Mediterranean

**DOI:** 10.3389/fmicb.2019.00858

**Published:** 2019-04-24

**Authors:** Isabel Marín-Beltrán, Jürg B. Logue, Anders F. Andersson, Francesc Peters

**Affiliations:** ^1^Institut de Ciències del Mar (CSIC), Barcelona, Spain; ^2^Department of Biology/Aquatic Ecology, Lund University, Lund, Sweden; ^3^Centre for Ecology and Evolution in Microbial Model Systems (EEMiS), Linnaeus University, Kalmar, Sweden; ^4^Science for Life Laboratory, Department of Gene Technology, KTH Royal Institute of Technology, Stockholm, Sweden

**Keywords:** atmospheric deposition, Saharan dust, anthropogenic aerosols, bacterial community composition, 454-pyrosequenciation, Mediterranean Sea

## Abstract

Atmospheric deposition is a source of inorganic nutrients and organic matter to the ocean, and can favor the growth of some planktonic species over others according to their nutrient requirements. Atmospheric inputs from natural and anthropogenic sources are nowadays increasing due to desertification and industrialization, respectively. While the impact of mineral dust (mainly from the Saharan desert) on phytoplankton and bacterial community composition has been previously assessed, the effect of anthropogenic aerosols on marine bacterial assemblages remains poorly studied. Since marine bacteria play a range of roles in the biogeochemical cycles of inorganic nutrients and organic carbon, it is important to determine which taxa of marine bacteria may benefit from aerosol fertilization and which not. Here, we experimentally assessed the effect of Saharan dust and anthropogenic aerosols on marine bacterioplankton community composition across a spatial and temporal range of trophic conditions in the northwestern Mediterranean Sea. Results from 16S rDNA sequencing showed that bacterial diversity varied significantly with seasonality and geographical location. While atmospheric deposition did not yield significant changes in community composition when all the experiments where considered together, it did produce changes at certain places and during certain times of the year. These effects accounted for shifts in the bacterial community’s relative abundance of up to 28%. The effect of aerosols was overall greatest in summer, both types of atmospheric particles stimulating the groups *Alphaproteobacteria, Betaproteobacteria*, and *Cyanobacteria* in the location with the highest anthropogenic footprint. Other bacterial groups benefited from one or the other aerosol depending on the season and location. Anthropogenic aerosols increased the relative abundance of groups belonging to the phylum *Bacteriodetes* (*Cytophagia, Flavobacteriia*, and *Sphingobacteriia*), while Saharan dust stimulated most the phytoplanktonic group of *Cyanobacteria* and, more specifically, *Synechococcus*.

## Introduction

Marine bacterioplankton play fundamental roles in the cycling of carbon. Photosynthetic bacteria (i.e., *Cyanobacteria*) take up CO_2_ and transform it into organic matter. Heterotrophic bacteria either remineralize parts of the organic carbon (OC) and thus release CO_2_ back into the atmosphere, or transform labile dissolved OC into recalcitrant OC that persists for long times in the ocean (Jiao et al., 2010). While microorganisms modify the composition of the OC pool, the composition of organic matter compounds and their availability to microbes strongly influence bacterioplankton activity and community composition (Kirchman et al., 2004; Jiao and Zheng, 2011; Logue et al., 2016). Apart from OC, there are other factors that control community structure, such as the bioavailability of inorganic nutrients that may limit bacterial growth and production (Kirchman et al., 2004).

In the oligotrophic Mediterranean Sea, as in the global ocean, large amounts of OC have been recorded in its surface waters during the summer stratification period (May–October). This phenomenon has been attributed to a “malfunctioning” of the microbial loop, brought forth by nutrient limitation or competition between prokaryotes and phytoplankton (Thingstad et al., 1997; Pinhassi et al., 2006). As a matter of fact, Mediterranean waters – especially during that stratification period – are depleted of inorganic nutrients. Hence, the growth and production of the marine planktonic community is predominantly limited by phosphorus (P) or P along with nitrogen (N); the latter co-limitation found mainly in the northwestern (NW) basin (Sala et al., 2006; Guadayol et al., 2009; Pulido-Villena et al., 2014). Nutrient limitation is more conspicuous in the open ocean than in coastal waters, which is illustrated by a mean annual chlorophyll concentration of ca. 0.25 μg l^-1^ (Gallisai et al., 2014; Gomes et al., 2015) as to 0.63 and 1.5 μg l^-1^ observed in waters adjoining rural (Guadayol et al., 2009) and urban (Arin et al., 2013; Romero et al., 2014) areas of the Catalan coast, respectively. In the NW Mediterranean, OC may also limit heterotrophic planktonic growth and production during periods of winter mixing (December–March), both in open ocean (Thingstad et al., 1997) and coastal waters (Pinhassi et al., 2006).

The deposition of particles from the atmosphere onto the Mediterranean Sea may, to some extent, alleviate the nutrient and OC shortage. The atmosphere of the NW Mediterranean experiences frequent inputs of total suspended particles from European and local sources but also from the Saharan desert (Guerzoni et al., 1999; Rodríguez, 2002). With deposition fluxes of total suspended particles of up to thousand times greater (up to 22 g m^-2^ d^-1^; Ternon et al., 2010) than during normal weather conditions (e.g., 15 mg m^-2^ d^-1^ in the eastern coast of Spain; Pulido-Villena et al., 2008), Saharan dust (SD) events can account for a large part of atmospheric inputs in the NW Mediterranean. This is especially true during the summer season (Querol et al., 2009). In addition, atmospheric background levels largely vary between urban (39 μg m^-3^; Barcelona; Pey et al., 2008) and rural (16 μg m^-3^; Montseny atmospheric station, located at the north-east of Spain; Querol et al., 2009) Mediterranean areas. The chemical composition of SD over the Mediterranean atmosphere, although variable depending on the source area, has been broadly characterized with the main chemical constituents being silicate and aluminum oxides (Moreno et al., 2006; Nava et al., 2012). To a lower extent, SD is also a source of N, P, OC, and metals to land and ocean (Guerzoni et al., 1999; Ridame and Guieu, 2002; Morales-Baquero et al., 2006). While N is thought to be soluble in seawater (and thus bioavailable), the fraction of leachable P from SD is highly variable (7–100%; Nenes et al., 2011; Stockdale et al., 2016). On the other hand, anthropogenic aerosols (AA) are more acidic compared to SD. This facilitates the leachability of P, organic compounds, and trace metals to seawater (Durrieu de Madron et al., 2011; Longo et al., 2014), being therefore more bioavailable to the plankton.

Marine bacterioplankton have been shown to benefit from both SD and AA (Pulido-Villena et al., 2008; Reche et al., 2009; Ternon et al., 2011; Marín et al., 2017b). However, their effect on the bacterial community composition remains poorly studied, with most of the previous work focusing on the effect of SD and using molecular techniques of low taxonomic resolution based on fluorescence *in situ* hybridization or denaturing gradient gel electrophoresis (Reche et al., 2009; Lekunberri et al., 2010; Marañón et al., 2010; Laghdass et al., 2012; Pulido-Villena et al., 2014). Only two recent studies have assessed the effect of AA on bacterial community composition (Guo et al., 2016; Rahav et al., 2016), but they were restricted to a single location at a given time of the year (during spring, offshore Crete). Results from these studies suggest that atmospheric deposition can selectively stimulate certain bacterial groups by providing specific conditions favorable to their growth. Hence, since each specific taxon or clade has its own growth requirements, variations in the proportion of nutrients supplied by aerosols (both natural and anthropogenically derived) may imply major changes in the composition of bacterioplankton assemblages. And because the concentration of both types of aerosols are expected to increase globally due to desertification and human activities (Duce et al., 2008; Reche et al., 2009; Durrieu de Madron et al., 2011), studying their effect on marine bacterial assemblages is of great global interest.

In this study, we assessed the relationship between atmospheric deposition of aerosols of different origin and bacterial community composition in the NW Mediterranean Sea by means of 16S rDNA pyrosequencing. To do so, we conducted six different experiments covering a spatial and a temporal gradient: on the one side, we tested the effect of aerosols in three locations that differed in their anthropogenic signature (coastal waters of Barcelona, coastal waters of Blanes – an area less affected by human impacts –, and offshore waters) during summer conditions; on the other side, sampling was done from winter to summer. In each experiment, we assessed the effect of SD and AA compared to non-amendment condition (i.e., controls). We hypothesized that aerosols will favor the stimulation of certain bacterial groups over others depending on the initial conditions, as the inorganic nutrients and OC concentration in the seawater would be different. More specifically, we hypothesized that (i) AA would yield a larger stimulation of heterotrophic bacteria, due to their higher content in soluble P and organic compounds; (ii) bacterial community composition of the urban location, especially in winter conditions, would be less disturbed by aerosols than in more oligotrophic locations, since bacteria may have access to nutrients from other sources in the former; and (iii) aerosols would decrease bacterial diversity by favoring the increase of opportunistic bacteria as a result of increased nutrient conditions.

## Materials and Methods

### Aerosol Collection

Total suspended particles were sampled at the Institut de Ciències del Mar of Barcelona (41.39° N, 2.20° E) and at the Centre d’Estudis Avançats of Blanes (41.68° N, 2.80° E) on days that Saharan particles arrived to the northeastern (NE) Iberian Peninsula (“SD” aerosols) or during normal meteorological conditions (“AA” aerosols). Total suspended particles were, thereby, collected on quartz fiber filters (Munktell; Falun, Sweden) by means of a CAV-A/mb high volume sampler (30 m^3^ h^-1^, 24 h; MCV; Barcelona, Spain), following the same protocol as in Marín et al. (2017a,b). SD and AA were classified based on advanced event warnings for the NE Iberian Peninsula^1^ and the element ratios criteria defined in the literature (Wedepohl, 1995; Migon et al., 2001; Guieu et al., 2010; Nava et al., 2012). Each filter served two purposes: one half was used for chemical composition analyses, while the other was employed as inoculum in the subsequent aerosol-addition experiments (see Supplementary Methods S1 for more details).

### Experimental Setup

#### Microcosms Setup

Water was collected either close to the coast of Barcelona (BCN) and Blanes (BLA) or offshore (OFF) the Balearic Islands in the NW Mediterranean Sea. Experiments were carried out in late summer of 2013 in Barcelona (SU-BCN), and during 2014 in winter and spring in Barcelona (WI-BCN and SP-BCN), spring and summer in Blanes (SP-BLA and SU-BLA), and in late summer offshore (SU-OFF) (Table 1). In Barcelona and Blanes, surface water (0.5 m) was collected in acid-cleaned carboys and transferred to the laboratory at the Institut de Ciències del Mar of Barcelona in less than 2 h. The SU-OFF experiment was performed on board of the research vessel *García del Cid*. Here, we collected 5 m-deep water using 10-l Niskin bottles mounted on a rosette frame. In all cases, immediately upon collection, the water was on site first sieved through a 150-μm-nylon mesh to remove macro-zooplankton. Water was distributed into 15-l acid-cleaned methacrylate containers (BCN, BLA) or 10-l acid-cleaned polypropylene carboys (OFF). Microcosms were incubated at *in situ* temperatures and subjected to *in situ* day-night cycles (see Table 1 for detailed information). Irradiance inside the containers was 225 μmol photons m^-2^ s^-1^, which is approximately the saturating irradiance reported at the Catalan coast for most of the year (Guadayol et al., 2009).

**Table 1:** Summary of the experimental setup.

	WI-BCN	SP-BCN	SU-BCN	SP-BLA	SU-BLA	SU-OFF
Date (experiment start)	25.02.2014	12.05.2014	18.09.2013	8.04.2014	30.06.2014	26.09.2014
Season	Winter	Spring	Summer	Spring	Summer	Summer
Location	Barcelona	Barcelona	Barcelona	Blanes	Blanes	Offshore
Latitude (°N)	41.38	41.38	41.38	41.67	41.67	39.55
Longitude (°E)	2.22	2.22	2.22	2.80	2.80	4.93
Bottom depth (m)	40	40	40	22	22	2655
SST (°C)	13.3	17.6	23.4	14.0	21.4	22.0
Light:dark (h)	11:13	14.5:09.5	12.5:11.5	13:11	15:09	12.5:11.5
TREATMENT	C, AA	C, AA, SD	C, AA, SD	C, AA, SD	C, AA, SD	C, AA, SD
Incubation time^∗^	4.92	3.93	4.38	1.90	2.90	1.90
Total # Samples	5	6	6	6	6	6


*The atmospheric treatment was evaluated using two duplicate containers either amended with an aerosol type or left unamended: C = control, AA = anthropogenic, SD = Saharan; SST = sea surface temperature; “Bottom depth” refers to the maximum depth at the sampling station. ^∗^Days since the aerosol addition.*

#### Amendments

Aerosol amendments to the experimental containers consisted of 0.8 mg l^-1^ of either SD or AA. Amendments of 0.8 mg l^-1^ are equivalent to a medium-high Saharan deposition event of 8 mg m^-2^ (a general value for SD events reported in the NE coast of Spain; Guerzoni et al., 1999) into a mixed layer water column of 10 m, which is approximately the depth of the thermocline during the stratification period in the NW Mediterranean (D’Ortenzio et al., 2005). In each experiment, another two containers were either not amended or amended with a blank filter processed identically as the filters with aerosols to verify that no fertilization effect could be attributed to the filters themselves. These two types of control showed no significant differences between them (Marín et al., 2017a). Thus, to simplify, we considered them duplicates and referred to them as controls (C). In all the experiments, each treatment was evaluated in duplicate containers (*N* = 2). As Saharan events over the NW Mediterranean are less frequent during winter, we used four containers amended with 0.8 mg l^-1^ of AA in WI-BCN (and no SD microcosms). In this experiment, we lost one of the controls, leaving one C and four AA samples (Table 1). Aerosols used for each experiment were collected at the correspondent location (i.e., Barcelona or Blanes), with the exception of the experiment carried out offshore; here, aerosols from Barcelona were used for the amendment, as the campaign was not long enough to collect and process the filters on board.

#### Sampling Collection

Samplings before and after the aerosol additions, as well as at the end of incubation time were conducted to determine the concentration of nitrate (NO_3_^-^), ammonium (NH_4_^+^), total inorganic phosphorous (TIP), total organic carbon (TOC), chlorophyll *a* (Chl), heterotrophic bacterial abundance (HBA), and heterotrophic bacterial production (HBP) (see Marín et al., 2017a,b for a full description of analyses thereof). Samples for bacterial composition analyses were collected at the end of the incubation period. To analyze bacterial community composition, 250 ml samples were filtered onto 0.2-μm polycarbonate filters (Durapore Membrane Filters, Millipore; Billerica, MA, United States). Filters were placed into sterile 2-ml cryogenic vials (Nalgene; Rochester, MN, United States) and stored immediately at -80°C until further processing.

### Bacterial Community Composition Analyses

#### Nucleic Acid Extraction

Bacterial DNA extraction was conducted following the procedure described in Schauer et al. (2000) with minor modifications. In brief, filters were submerged in lysis buffer (40 mM EDTA, 50 mM Tris [pH = 8.3], 0.75 M sucrose) and - after lysozyme had been added at a final concentration of 1 mg ml^-1^ - incubated at 37°C for at least 45 min under slight movement. Upon adding proteinase K (0.2 mg ml^-1^ final concentration) and sodium dodecyl sulfate (10% v/v), samples were again incubated at 55°C for at least 1 h. Approximately 750 μl of lysate was extracted from the filters, mixed twice with 750 μl of a mixture of phenol-chloroform-isoamyl alcohol (25:24:1, pH = 8), respectively, and once with 750 μl of chloroform-isoamyl alcohol (24:1). The aqueous phase was recovered and concentrated into Amicon Ultra-15 Centrifugal Filter Units (Millipore; Billerica, MA, United States) to approximately 250 μl, using a Sigma 3-16KL centrifuge (Sigma; Osterode am Harz, Germany) operating at 3000 rpm, and washed three times with 2 ml of supra-pure filtered (0.2 μm) water (milli-Q) to a final volume of 100-200 μl. The final extract was kept at -80°C.

#### PCR Amplification and Pyrosequencing

PCR amplification and pyrosequencing were carried out at the Research and Testing Laboratory of Lubbock^2^ (Lubbock, TX, United States). The bacterial hypervariable regions V1, V2, and V3 of the 16S rRNA gene were PCR amplified, using a forward and a reverse fusion primer 28F (5′-GAGTTTGATCNTGGCTCAG-3′) and 519R (5′-GTNTTACNGCGGCKGCTG-3′) (Handl et al., 2011), respectively. Reactions were performed on ABI Veriti thermocyclers (Applied Biosytems; Carlsbad, CA, United States) according to the following thermal profile: 95°C for 5 min, 35 cycles of 94°C for 30 s, 54°C for 40 s, and 72°C for 1 min, and finalized by one cycle at 72°C for 10 min. Sequencing was performed on a 454 GS-FLX+ system (454 Life Sciences). See Supplementary Methods S2 for a more detailed description.

#### Sequence Analyses

Pyrosequences were processed in QIIME (v 1.6) (Caporaso et al., 2010). After de-multiplexing and a first quality check, sequences were between 125 and 600 bp long, showed a quality score > 25, contained no more than two mismatches in the primer sequences, and no homopolymers longer than 6 bp. Denoised centroids and singletons were clustered into operational taxonomic units (OTUs) at a sequence identity level of 97% using UCLUST (Edgar, 2010). Representative sequences were aligned according to the SILVA (Quast et al., 2013) alignment (release 108). The remaining sequences were again aligned and taxonomy was assigned according to the SILVA alignment (release 123) (see Supplementary Methods S2 for a more detailed description). Finally, pyrosequences that were either assigned as Archaea, Eukaryota, or uncertain, or contained fewer than two reads, were removed from the final dataset. Sequence data can be found at the GenBank database under accession numbers SAMN05914888-SAMN05914893, SAMN05914900-SAMN0591414910, SAMN05914922-SAMN05914933, and SAMN05914936-SAMN05914941 (see Supplementary Table S1 to see the equivalent number for each sample).

### Statistical Analyses

We calculated the increase/decrease of the variables NO_3_^-^, NH_4_^+^, TIP, TOC, Chl, HBA and HBP in the aerosol-amended microcosms (AA, SD) with respect to the controls by means of the aerosol-induced ratio (AIR), following the procedure of Marín et al. (2017a). This ratio considers the maximum value reached by a variable in the aerosol-amended microcosms during the incubation period, and compares it with the maximum value reached by that variable in the controls. In addition, for each of these variables, we considered all data during the incubation period and conducted non-parametric Wilcoxon tests (Wilcoxon, 1945) to compare how these variables behaved in the different treatments. Data from the winter experiment were not considered, since there were no Saharan samples in this experiment.

For bacterial community composition, we evaluated the differences in read counts between aerosol types within experiments using the DESeq2 package (Love et al., 2014). Analyses were done at individual OTU level and at class level. *P*-adjusted values according to the false discovery rate (Benjamini and Hochberg, 1995) were calculated from a negative binomial distribution and significant differences were considered when *p*-adjusted was below an alpha cutoff value of 0.1. In the experiments where significant differences were found, either at OTU or class level, we calculated the percentage of the relative abundance of these groups in a given treatment (AA/SD). This is interpreted as the change in the community structure yielded by aerosols. This was calculated as the sum of the relative abundance of the taxonomic groups that significantly changed in that treatment – compared to another – and dividing it by the total (relative) abundance. The latter was calculated as the sum of all the relative abundances of each bacterial group present in that particular treatment. These results were plotted using the superheat package of the R software (Barter and Yu, 2017). We then conducted canonical correspondence analysis (CCA) to investigate the relationship between the bacterial community composition (at OTU or class level) and the biogeochemical variables (NO_3_^-^, TIP, TOC, Chl, HBA and HBP) measured at the end of the incubation. Only the OTUs/classes for which significant differences between treatments were found were plotted. Data were previously log-transformed. The ggvegan package (Wickham, 2009) was used for this purpose.

Alpha-diversity in the samples was calculated by means of the Chao 1 richness index and the Shannon index. The latter considers the combined richness and evenness within communities (Oksanen et al., 2008). The number of reads per sample was previously normalized by dividing the total number of reads in each sample by the lowest number of reads in the matrix. In order to compare samples from experiments with the same characteristics, the Chao 1 and Shannon indexes were calculated considering all the samples except those from the winter experiments to look for differences between treatments (*N* = 30). To compare differences between seasons, we considered the experiments carried out in Barcelona (WI-BCN, SP-BCN and SU-BCN; *N* = 17), and to compare the diversity between locations, we considered the experiments carried out in summer (SU-BCN, SU-BLA and SU-OFF; *N* = 18) (Table 1). To test whether Chao 1 and Shannon varied significantly with respect to treatment, season, or location, permutational multivariate analyses of variance (PERMANOVAs) based on Bray-Curtis distance (9999 permutations; *p* < 0.05) were computed. *p*-values were adjusted according to the false discovery rate in order to correct for multiple testing (Benjamini and Hochberg, 1995). These analyses were computed using the vegan package (v 3.2.4; Oksanen et al., 2008).

## Results

### Aerosol Composition and Release of Nutrients Into the Seawater

Results of aerosol chemical composition and nutrients released by the aerosols in the microcosms are shown and discussed elsewhere (Marín et al., 2017b). Concentrations of NO_3_^-^, NH_4_^+^, TIP, and TOC before the aerosol addition, after the addition, and at the end of the incubation period for each experiment can be found in the Supplementary Material (Supplementary Figure S1). Concentrations of inorganic nutrients and TOC measured before the aerosol additions were highly variable depending on the season and location (Table 2 and Supplementary Figure S1). Briefly, NO_3_^-^, NH_4_^+^, and TIP showed higher values in spring, especially in Barcelona; while TOC showed the highest values at the start of the summer experiment in Barcelona (SU-BCN). After the aerosol addition, NO_3_^-^ showed an average increase ratio of 2.79 ± 1.27 in the microcosms amended with AA (from herein after referred as AA microcosms) compared to the controls (C), and of 1.91 ± 1.12 in the microcosms amended with SD (from herein after referred as SD microcosms) (Table 2). TIP showed an increase ratio of 1.17 ± 0.36 in the microcosms amended with AA, and of 1.26 ± 0.66 in the microcosms amended with SD. NH_4_^+^ increased by 6.40 ± 6.83 and 2.46 ± 1.40 in the AA and SD microcosms, respectively. TOC increased by, on average, a ratio of 1.05 ± 0.07 in the AA microcosm, while it decreased in the SD compared to the C, with a ratio of 0.98 ± 0.04. Considering all data measured during the incubation period after the aerosol addition (*N* = 156), the Wilcoxon test showed that NO_3_^-^ was significantly higher in the AA treatment compared to the C (*p* < 0.0001) and the SD (*p* = 0.0005), and in the SD than in the C (*p* < 0.0001). NH_4_^+^ and TIP were significantly higher in the AA treatment than in the controls (*p* = 0.0024 and *p* = 0.0373; respectively), while no significant differences were detected for TOC.

**Table 2:** Biogeochemical data measured before the aerosol addition (T0) in the AA and SD microcosms. The aerosol-induced ratio (AIR) shows the increase/decrease of a given variable in the AA and SD microcosm (averaged for the replicates, *N* = 2) after the addition, compared to the controls.

	WI-BCN (AA)	SP-BCN (AA)	SP-BCN (SD)	SU-BCN (AA)	SU-BCN (SD)	SP-BLA (AA)	SP-BLA (SD)	SU-BLA (AA)	SU-BLA (SD)	SU-OFF (AA)	SU-OFF (SD)
NO_3_^-^ (T0) (μM)	0.92 ± 0.07	2.38 ± 0.30	2.44 ± 0.08	0.78 ± 0.55	0.48 ± 0.02	1.39 ± 0.16	1.17 ± 0.16	0.34 ± 0.02	0.42 ± 0.10	0.34 ± 0.00	0.34 ± 0.00
NO_3_^-^ (AIR)	1.92 ± 0.65	1.58 ± 0.10	0.75 ± 0.02	2.43 ± 1.14	1.83 ± 0.17	2.72 ± 0.04	1.55 ± 0.07	4.10 ± 0.33	1.57 ± 0.05	4.89 ± 0.18	3.88 ± 0.07
NH_4_^+^ (T0) (μM)	0.27 ± 0.03	0.5 ± 0.05	0.52 ± 0.00	0.12 ± 0.10	0.05 ± 0.00	0.02 ± 0.00	0.03 ± 0.00	0.02 ± 0.00	0.11 ± 0.12	0.16 ± 0.00	0.16 ± 0.00
NH_4_^+^ (AIR)	3.01 ± 1.63	3.56 ± 0.22	2.08 ± 0.29	4.67 ± 5.03	4.45 ± 1.10	21.63 ± 0.15	1.94 ± 0.10	7.01 ± 0.8	2.00 ± 2.55	1.93 ± 0.55	1.84 ± 0.26
TIP (T0) (μM)	0.05 ± 0.00	0.26 ± 0.00	0.23 ± 0.00	0.03 ± 0.00	0.03 ± 0.00	0.14 ± 0.00	0.15 ± 0.00	0.06 ± 0.01	0.08 ± 0.012	0.04 ± 0.00	0.04 ± 0.00
TIP (AIR)	1.13 ± 0.16	0.92 ± 0.05	0.87 ± 0.05	1.95 ± 0.07	2.48 ± 0.39	1.07 ± 0.03	0.96 ± 0.01	0.85 ± 0.06	0.91 ± 0.129	1.14 ± 0.10	1.11 ± 0.07
TOC (T0) (μM)	67.72 ± 2.64	85.97 ± 0.98	86.10 ± 1.90	93.15 ± 4.17	98.2 ± 2.69	70.16 ± 0.74	69.69 ± 1.28	81.07 ± 2.02	83.94 ± 0.89	81.69 ± 0.00	81.69 ± 0.00
TOC (AIR)	1.13 ± 0.06	1.01 ± 0.03	1.01 ± 0.02	1.00 ± 0.09	0.97 ± 0.10	1.07 ± 0.00	0.99 ± 0.01	1.03 ± 0.11	0.947 ± 0.02	1.03 ± 0.01	0.98 ± 0.03
Chl (T0) (μg l^-1^)	1.90 ± 0.00	1.22 ± 0.00	1.22 ± 0.00	0.44 ± 0.00	0.44 ± 0.00	0.26 ± 0.00	0.26 ± 0.00	0.27 ± 0.00	0.27 ± 0.00	0.06 ± 0.00	0.06 ± 0.00
Chl (AIR)	1.06 ± 0.05	1.40 ± 0.05	1.17 ± 0.01	2.15 ± 0.22	2.24 ± 0.23	1.42 ± 0.13	1.15 ± 0.01	1.12 ± 0.16	1.01 ± 0.01	1.15 ± 0.35	0.75 ± 0.07
HBA (T0) ( × 10^5^ cell ml^-1^)	5.06 ± 0.00	11.5 ± 0.00	11.5 ± 0.00	6.63 ± 0.00	6.63 ± 0.00	4.70 ± 0.00	4.70 ± 0.00	8.03 ± 0.00	8.03 ± 0.00	4.87 ± 0.00	4.87 ± 0.00
HBA (AIR)	1.00 ± 0.00	1.05 ± 0.18	1.20 ± 0.07	1.38 ± 0.02	1.49 ± 0.12	1.13 ± 0.21	0.97 ± 0.05	1.11 ± 0.15	1.00 ± 0.01	0.94 ± 0.01	1.05 ± 0.08
HBP (T0) (μg C l^-1^ d^-1^)	4.53 ± 0.00	1.19 ± 0.00	1.19 ± 0.00	1.91 ± 0.00	1.91 ± 0.00	3.51 ± 0.00	3.51 ± 0.00	3.79 ± 0.00	3.79 ± 0.00	0.08 ± 0.00	0.08 ± 0.00
HBP (AIR)	1.06 ± 0.19	1.40 ± 0.07	1.14 ± 0.00	1.84 ± 0.39	1.87 ± 0.35	1.97 ± 0.18	1.17 ± 0.00	1.98 ± 0.03	1.40 ± 0.08	3.26 ± 0.08	1.07 ± 0.17


### Effect of Aerosols on Chlorophyll and Bacterial Abundance and Production

As with nutrients, concentrations of Chl, HBA, and HBP at the start of the experiments are shown in Table 2 and Supplementary Figure S2. Supplementary Figure S2 also shows the concentrations after the addition and at the end of the incubation period for each experiment. After the addition, Chl increased by, on average, 1.38 ± 0.96 in the AA microcosms compared to the C, and by 1.33 ± 0.33 in the SD microcosms. HBA showed an overall low increase of 1.09 ± 0.16 in the AA and 1.14 ± 0.21 in the SD microcosms, compared to the C. HBP increased by 1.80 ± 0.75 in the AA microcosms, and by 1.33 ± 0.33 in the SD microcosms. Non-parametric Wilcoxon tests carried out with samples from all the experiments but the winter one (*N* = 156), showed that HBP was significantly higher in the AA than in the C microcosms (*p* = 0.0362). A detailed discussion on the behavior of Chl, HBA, and HBP in these experiments can be found in previous publications (Marín et al., 2017a,b).

### Bacterial Community Dynamics

We analyzed bacterial community composition in 35 samples across the six experiments (WI-BCN, SP-BCN, SU-BCN, SP-BLA, SU-BLA, SU-OFF; Table 1). Using 454-pyrosequenciation, we obtained 208,106 high quality 16S rRNA gene sequences clustering into 2,842 operational taxonomic units (OTUs) at a sequence similarity of 97%. The samples contained on average 5,946 sequences and in all cases no less than 1,000 sequences.

When OTUs were grouped into taxonomic classes that accounted for >1% of the total relative abundance, we found 6 groups that dominated in most of the samples: *Alphaproteobacteria, Cyanobacteria* (mainly from the genus *Synechoccocus*), *Deltaproteobacteria*, *Flavobacteriia, Gammaproteobacteria*, and *Sphingobacteriia* (Supplementary Figure S3). *Alphaproteobacteria* constituted >45% of the total relative abundance in all the samples. *Cyanobacteria* were the second most abundant group, accounting for more than 25% in the SP-BLA samples and in the samples amended with SD in SU-BLA and SU-OFF. The contribution of *Deltaproteobacteria* accounted for ca. 10% of the SU-BCN and SU-BLA samples, constituting >15% of the abundance in the controls of SU-BCN. *Flavobacteriia* were more frequent in the samples from Barcelona, especially in the spring samples (SP-BCN), accounting for up to 28% of the total relative abundance in the samples amended with SD. *Gammaproteobacteria* accounted for more than 10% of the relative abundance in the samples of WI-BCN, in the controls of SP-BCN and SU-BCN, and in the controls and AA samples of the offshore experiment. *Sphingobacteriia* were most abundant in SP-BCN, especially in the AA treatment, where they accounted for 11% of the total relative abundance. The contribution of the remaining taxonomic groups was less than 1%, but significant differences were found in some of these groups between experiments (data not shown) and between treatments within a given experiment (see section “Effect of Aerosols on Bacterial Community Composition”).

#### Effect of Aerosols on Bacterial Community Composition

**FIGURE 1:**
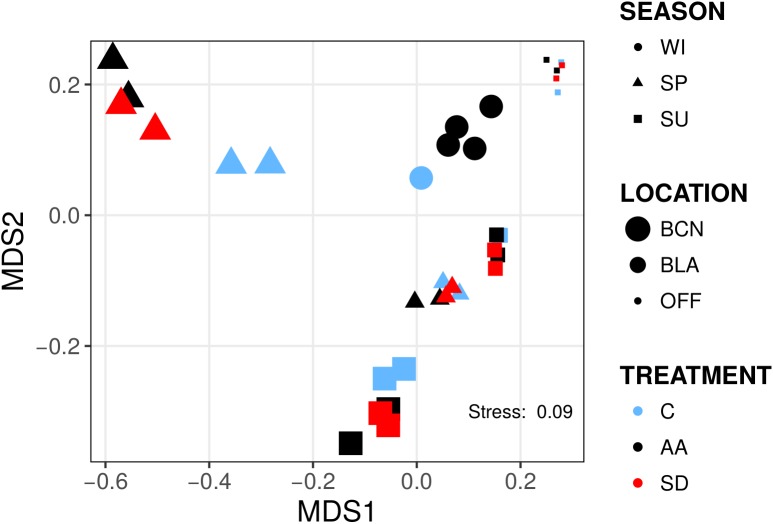
Non-metric multidimensional scaling (NMDS) representation of bacterial communities from the six experiments. Samples are shaped-coded according to season, sized-coded according to location, and color-coded according to treatment (legend on the right). NMDS ordination was derived from pairwise Bray–Curtis distances. Each symbol corresponds to one sample (*N* = 35).

**FIGURE 2:**
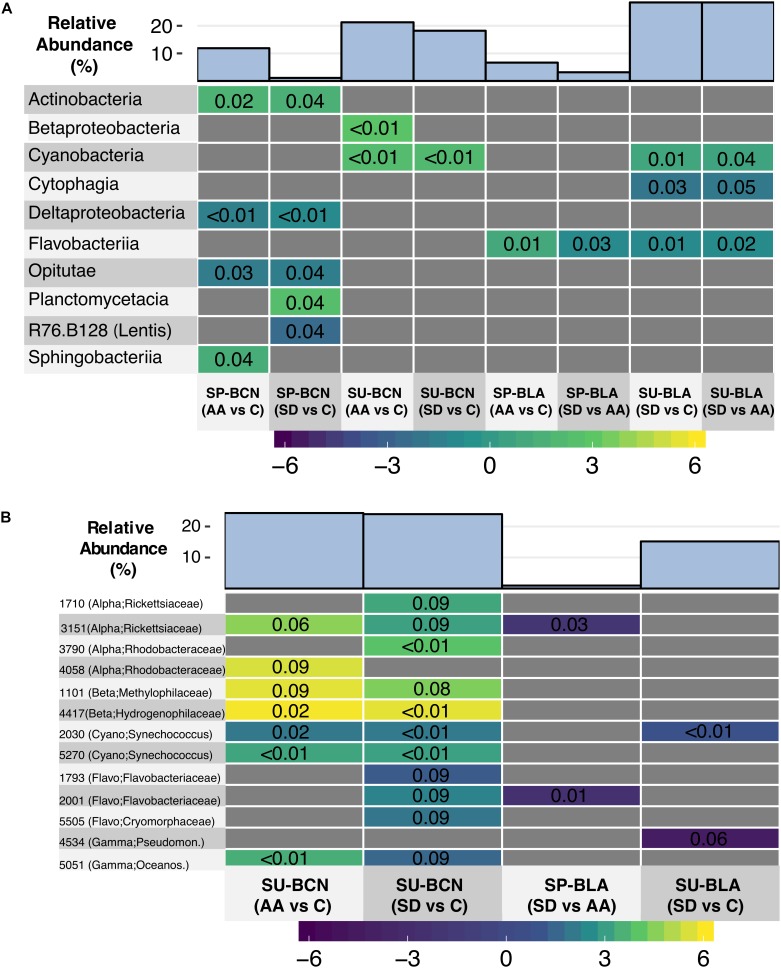
Heatmaps showing the change in the relative abundance of marine bacteria classes **(A)** and OTUs **(B)** in a given treatment compared to another – specified within the parenthesis – within experiments. Note that results are only shown for those experiments where significant differences were found between treatments. The colored scale indicates the Log_2_-Fold change, while the *p*-value is indicated inside the colored squares. The sum of the relative abundance (in %) of the classes/OTUs that changed significantly with respect to the total (relative) abundance in a given treatment is shown on the top. The phylogenetic composition of each OTU is shown in parenthesis. Abbreviations: Alpha = *Alphaproteobacteria*; Beta = *Betaproteobacteria*; Cyano = *Cyanobacteria;* Flavo = *Flavobacteriia*; Gamma = *Gammaproteobacteria;* Lentis = *Lentisphaerae;* Oceanos = *Oceanospirillaceae*; Pseudomon = *Pseudomonadaceae*.

Bacterial communities were distinct from one another in composition and clustered mainly according to location and time of the year as a Non-metric Multidimensional Scaling ordination depicted (Figure 1). We found significant differences between locations and times of the year at different taxonomic levels; however, this is out of the scope of this article and instead we will focus on the changes attributed to aerosols. Whereas analyzing differences in the relative abundance of bacterial groups between treatments (AA, SD, and C) did not yield significant differences when all the experiments were considered together, differences were found when the analyses were conducted within experiments. At class level, we found significant differences in the experiments SP-BCN, SU-BCN, SP-BLA, and SU-BLA (Figure 2A). In SP-BCN, aerosol amendments brought about different changes in the relative abundance of several groups (compared with the controls), which accounted for 12 and 1% of the total relative abundance in the AA and SD microcosms, respectively (Figure 2A, upper part). In SU-BCN, the significant increase of *Betaproteobacteria* (in AA) and *Cyanobacteria* (in AA and SD) with added aerosols constituted changes of 21 and 18% in abundance in AA and SD, respectively. In SP-BLA, the changes in abundance were smaller. Here, the increase in *Flavobacteriia* in the AA treatment with respect the C and the SD drove changes of 7 and 3% in bacterial abundance, respectively. In SU-BLA, SD yielded an increase in the relative abundance of *Cyanobacteria* significantly higher than the other treatments (C, AA). On the contrary, the groups *Cytophagia* and *Flavobacteriia* were significantly less abundant in the SD microcosms compared to the C and the AA. These groups accounted for large changes in the total abundance of SD amended communities (28%).

Analyses at OTU level showed significant differences in the experiments SU-BCN, SP-BLA, and SU-BLA (Figure 2B). In SU-BCN, changes in the bacterial community attributed to the aerosol addition accounted for 24% of the total relative abundance in both AA and SD treatments. A number of OTUs belonging to *Betaproteobacteria* and *Cyanobacteria* (*Synechococcus*) were significantly more abundant in both amended treatments than in C. These results were in agreement with the changes observed at class level (Figure 2A). One *Alphaproteobacteria* OTU belonging to the family *Rickettsiaceae* and another one from the family *Rhodobacteraceae*, were significantly more abundant in the AA compared to the C microcosms, and the relative abundance of two OTUs of each family was higher in the SD compared to the C microcosms (Figure 2B). Both aerosols also yielded an increase in the relative abundance of one OTU belonging to the family *Oceanospirillaceae* (*Gammaproteobacteria*), and SD produced an increase in three OTUs from the class *Flavobacteriia*. In SP-BLA, one OTU belonging to the family *Rickettsiaceae* and another one from the family *Flavobacteriaceae*, were significantly less abundant in the SD treatment than in the AA, accounting for minor changes in these samples of 1% of the total abundance. In SU-BLA, similar to what observed at class level, one OTU of *Synechococcus* was significantly stimulated in the SD treatment, compared to the C. In contrast, one OTU belonging to the family *Pseudomonadaceae* (*Gammaproteobacteria*) was less abundant in the SD samples than in the C. The total change in the community structure attributed to SD was 15%.

**FIGURE 3:**
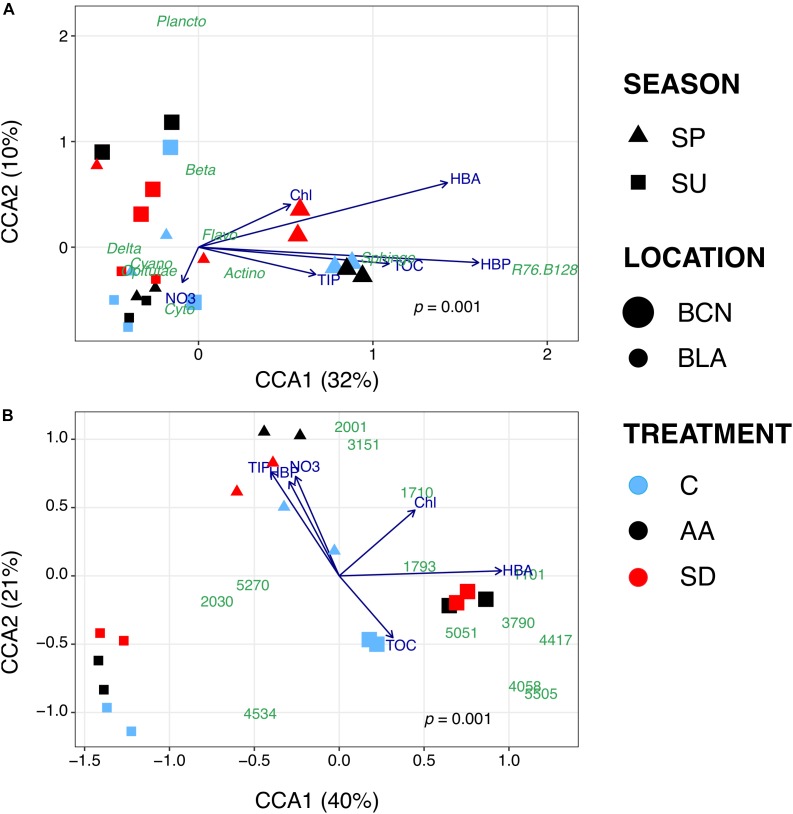
Bi-plot visualization of the CCAs carried out at class **(A)** and OTUs **(B)** level. Symbols represent bacterial communities in the experimental samples. Symbols are shaped-coded according to season, sized-coded according to location, and color-coded according to treatment (legend on the right). Each symbol represents one sample. Note that results are only shown for those experiments where significant differences were found between treatments. Abbreviations: Actino = *Actinobacteria*; Beta = *Betaproteobacteria*; Cyano = *Cyanobacteria;* Cyto = *Cytophagia*; Delta = *Deltaproteobacteria*; Flavo = *Flavobacteriia*; Plancto = *Planctomycetacia*; Sphingo = *Sphingobacteriia*.

To figure out why certain taxonomic groups were more abundant in some treatments compared to others, we carried out CCAs. In these analyses, we included the bacterial groups (Figure 3A)/OTUs (Figure 3B) that showed significant changes, and data of the biogeochemical variables measured at the end of the incubation time in each sample. At class level (*p* = 0.001; 42% of the variance explained by the selected variables; Figure 3A), samples from the SP-BCN experiment were separated from the other samples by the first component (CCA1). The plot shows samples from SP-BCN closer to most of the biogeochemical variables studied, pointing out that these samples contain higher concentrations of these variables. More precisely, AA samples of SP-BCN appear close to TIP, TOC, and *Sphingobacteriia* - which were more stimulated in this treatment (Figure 2A). A CCA constructed only with the samples from SP-BCN confirmed these results, *Actinobacteria* located also closer to the AA samples, NO_3_^-^, and HBP (data not shown). Samples from the other experiments were divided by the second component (CCA2), which was almost parallel to NO_3_^-^ concentration. The samples from SU-BCN had in general low NO_3_^-^, except one of the controls (Figure 3A and Supplementary Figure S1). Instead, SU-BCN samples presented a higher proportion of *Betaproteobacteria* than samples from Blanes, the latter located on the bottom-left corner of the plot. Samples from Blanes presented in general a higher NO_3_^-^ concentration, especially the AA. In agreement with results from DESeq2, the SD samples from SU-BLA were closer to *Cyanobacteria* than the AA and the C samples of the same experiment, while *Cytophagia* appeared closer to the C and the AA samples.

The CCA plot at OTU level (*p* = 0.001; 61% of the variance explained by the selected variables; Figure 3B) separated the samples from SU-BCN from the samples of Blanes by their content in HBA, which followed the first component. Samples from SU-BCN presented higher HBA (Supplementary Figure S2B and Figure 3B). In this plot, the differences between treatments in this experiment became more apparent. The AA and SD samples appeared close to several OTUs that were significantly more abundant in these samples (Figure 2B, 3B) than in the controls. HBA was also higher in the amended samples. These results suggested that these OTUs, belonging mainly to the groups *Rhodobacteraceae, Betaproteobacteria* and *Oceanospirillaceae*, were among the main contributors to HBA in these samples. OTUs belonging to the families *Flavobacteriaceae* and *Rickettsiaceae*, instead, appeared closer to Chl and HBP. The second component divided the samples from Blanes. Samples from the SP-BLA experiment were more abundant in NO_3_^-^, TIP and HBP than samples from SU-BLA (Figure 3B and Supplementary Figures S1A,C, S2C). This is especially true for the AA samples, which were also significantly more abundant in *Flavobacteriia* than the other samples, either at class (C and SD) or OTU (SD) level (Figure 2). Instead, the content of TOC was higher in the SU-BLA than in the SP-BLA samples (Figure 3B and Supplementary Figure S1D). Comparing the samples from SU-BLA, *Cyanobacteria* as a group (Figure 3A) and, more particularly, OTUs 2030 and 5270 – the second and the fifth most abundant overall –, belonging to *Synechococcus*, appeared closer to the SD than to the C and the AA samples (Figure 3B).

#### Effect of the Environmental Variables on Alpha-Diversity

**FIGURE 4:**
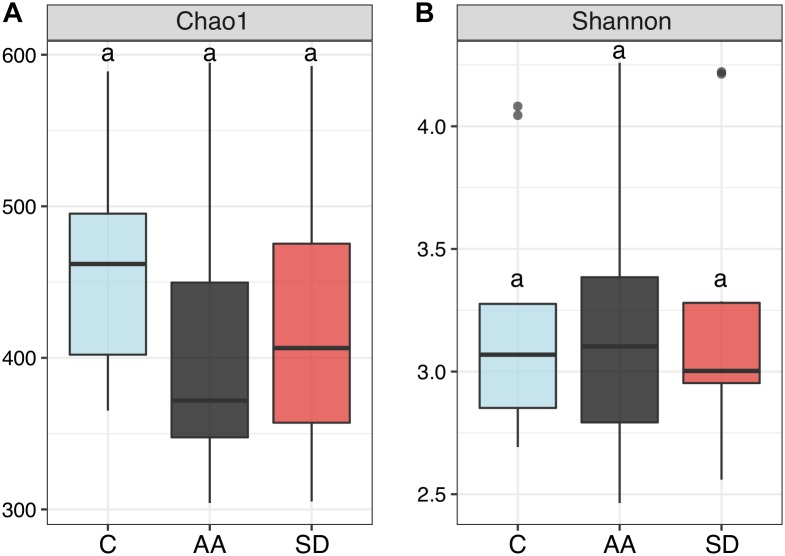
Box-plot representation of diversity indexes comparing the different treatments (C, AA, SD) in all the experiments but the winter one (*N* = 30). Diversity was measured by **(A)** Chao 1 index, and **(B)** Shannon index. The boxes indicate median and quartile values, while the whiskers indicate the range (minima and maxima). Same letter indicates no significant differences (PERMANOVA, *p* > 0.05) found between treatments.

We measured alpha-diversity to assess differences in bacterial community structure between the controls and the communities amended with AA or SD. Richness, estimated by the Chao 1 index, was the highest in the C samples (Figure 4A), but there were no significant differences between treatments (*p* = 0.2750). The Shannon index was similar in all the treatments (Figure 4B; *p* = 0.9921). Diversity indexes varied significantly between seasons and locations, though. In Barcelona, the Shannon index was significantly higher in spring than in winter (*p* = 0.0032) and summer (*p* = 0.0029) (Supplementary Figure S4). Comparing the three locations, the highest diversity was found in Barcelona. The Chao 1 estimator was significantly higher in Barcelona than offshore (*p* = 0.01; Supplementary Figure S5A). Shannon was significantly higher in Barcelona than in Blanes (*p* = 0.0022) and offshore (*p* = 0.0026), and significantly higher in Blanes than offshore (*p* = 0.0041) (Supplementary Figure S5B).

## Discussion

The main objective of this study was to evaluate the effect of different types of atmospheric particles on bacterial community composition in surface waters of the NW Mediterranean. In the area, the structure of bacterioplankton is variable in space (Schauer et al., 2000; Nogales et al., 2007), although the degree of variability is sometimes small (Acinas et al., 1997). Seasonality has also been suggested to be a predominant factor modulating the structure of bacterioplankton in the NW Mediterranean (Schauer et al., 2003; Pinhassi et al., 2006; Alonso-Sáez et al., 2007). Thus, in order to test for effects of atmospheric particles on bacterioplankton composition, we took into account the variability in both space and time. Although the ideal situation always is to do all experiments at all times and locations, this is rarely logistically possible and it was not feasible here owing to the limited timeframe needed to avoid longer-term inter-annual environmental changes.

Further caution is needed regarding a number of issues. Although a minimum of three replicates per experimental condition guarantees statistical robustness, experiments with medium to large containers and several different treatments rarely allow for such logistics. However, replicate containers behaved very similarly (Supplementary Figure S6), providing confidence in the results. Incubation times for the individual experiments could not always be exactly the same (e.g., the experiment carried out offshore lasted less than planned due to weather conditions). We have also taken this aspect into account when interpreting our results, since the temporal interval between aerosol amendment and response is a factor when evaluating bacterial community composition and diversity. For example, in the DUNE 1 experiment performed in the western Mediterranean, no noticeable differences between samples amended with SD and the controls were found sampling 8 days after seeding (Laghdass et al., 2011), whereas significant changes in the abundance of a few OTUs were observed in a similar mesocosm experiment sampled at a higher temporal resolution (DUNE-R; Pulido-Villena et al., 2014).

Despite these issues, to the best of our knowledge, this is the most comprehensive experimental set of data available addressing the effect of different types of aerosols on bacterial communities across a range of temporal and spatial scales. In agreement with previous studies, bacterial diversity varied significantly with season (Schauer et al., 2003; Alonso-Sáez et al., 2007; Pinhassi et al., 2006) and location (Schauer et al., 2000; Nogales et al., 2007), while atmospheric particles yielded major changes in bacterial assemblages at certain locations and times of the year.

### Diversity Changes Across Space and Time

We compared the effect of aerosols on bacterial diversity at locations with different anthropogenic footprint and at different times of the year. Since higher ambient nutrient pulses indicate a chance for opportunistic bacteria to overgrow other bacterial groups, we expected to find lower diversity during more eutrophic conditions. In summer, when we have data for all three locations, alpha diversity was highest in Barcelona, the most anthropogenically altered site and, thus, the most eutrophic. Although this may seem initially contradictory, these results agree with those found by Schauer et al. (2000) along the Catalan coast. Nogales et al. (2007) also found a more diverse bacterial community in a highly impacted marina from a coastal area in Mallorca (Spain), compared to an assembly sampled in a less impacted bay. Similarly, we found a clear pattern in alpha-diversity from highest in spring, to lower levels in winter and in summer, with decreasing ambient nutrient concentration (Supplementary Figure S1). No data in terms of bacterial diversity have been reported before in Barcelona at different times of the year. In Blanes, a lower diversity in spring compared to other seasons was previously observed (Alonso-Sáez et al., 2007), showing that, even within a season, initial conditions may vary substantially and affect bacterial community composition. These results would suggest that our initial hypothesis that oligotrophy favors the increase in diversity may be wrong. Perhaps, on the contrary, it may indicate that the large nutrient concentrations are not pulsed but rather continuous (Romero et al., 2012; Guillén et al., 2018) actually stabilizing water conditions and providing more different taxa the opportunity to develop evenly. In a study carried out at the Red Sea, Jessen et al. (2013) found out an increase in bacterial diversity associated to corals after eutrophication. In any case, this is an issue that needs to be explored in further studies.

Similarly, we expected that both types of aerosols, by providing more nutrients, would benefit opportunistic bacteria and thus decrease diversity. In this case, the high variability shown in Chao 1 and Shannon indexes prevents us from finding a clear pattern in the net diversity with respect to the aerosol treatment. This would indicate that the effect of aerosol amendment, albeit altering the proportion of some bacterial taxa, is not strong enough to substantially alter significantly holistic parameters of system structure, such as the diversity. In previous experiments in which SD was added to planktonic communities, no significant differences in bacterial diversity were found either (Reche et al., 2009; Pulido-Villena et al., 2014). It has also to be taken into account that we added realistic amounts of aerosols to the experimental microcosms, what makes difficult to observe large changes. Larger amendments should indeed alter the diversity. Also, the variability of initial conditions increases the uncertainty and points to the need to account for it with experiments that cover a range of conditions including locations and times of the year, as we started to do.

### Effect of Aerosols on the Bacterial Community

In the present study we are focusing, however, on the effect of atmospheric particles on bacterial communities. In this regard, our results showed that SD and AA triggered substantial changes in the composition of bacterial communities at given locations and times of the year in the NW Mediterranean Sea. Overall, the highest changes observed occurred in summer. This is in agreement with previous findings showing a larger response of bacteria to aerosols during most oligotrophic conditions (Marañón et al., 2010; Teira et al., 2013; Marín et al., 2017b). These differences observed between seasons are expected to be even higher in nature, since SD deposition events are more frequent and of larger magnitude during the summer season (Querol et al., 2009). While a potential toxic effect of aerosols on *Cyanobacteria*, and more particularly *Synechococcus*, has been previously discussed (Paytan et al., 2009; Marañón et al., 2010; Marín et al., 2017a), our results showed an overall increase of this group with aerosols in summer, especially with SD amendments. *Cyanobacteria* as a group, and more specifically *Synechococcus*, were indeed one of the principal groups favored by both types of aerosols (SU-BCN) or only by SD (SU-BLA). The increase of *Cyanobacteria* with aerosols during summer may be attributed to their advantage to outcompete larger phytoplankton cells in the uptake of nutrients during oligotrophic conditions, since they have a larger surface:volume ratio (Raven, 1998). In the SU-BLA experiment in particular, *Cyanobacteria* were also significantly more abundant in the SD than in the AA samples, while we observed the opposite pattern for nanoeukaryotes and diatoms (data not shown). *Betaproteobacteria* were also stimulated by both aerosol particles during summer in Barcelona (SU-BCN). In agreement with these results, Teira et al. (2013) reported an increase in *Betaproteobacteria* in response to urban rainwater additions.

In Blanes, major changes were observed for the group *Flavobacteriia*, which were either stimulated by AA (SP-BLA) or disfavored by the addition of SD (SU-BLA). *Flavobacteriia* as a group and certain OTUs within showed a correlation with Chl and NO_3_^-^ (Figure 3). That suggests that this group may have benefited from the increase of Chl observed in the AA treatment (Table 2; Supplementary Figure S2A). *Cytophagia* and *Sphingobacteriia*, two groups belonging also to the phylum *Bacteroidetes*, were more stimulated by AA compared to SD as well. *Sphingobacteriia* appeared close to TOC and TIP in the CCA, while *Cytophagia* was closer to NO_3_^-^ (Figure 3A). All these nutrients showed generally higher concentrations in AA microcosms. Thus, the higher content in nutrients and Chl in the AA samples may yield either a direct or an indirect effect on the members of *Bacteroidetes.* This is in agreement with studies showing an increase in *Bacteroidetes* in response to rainwater additions (Teira et al., 2013), as well as after algal blooms (Wietz et al., 2010). Regarding *Gammaproteobacteria*, this group has frequently been associated with algal blooms (Lebaron et al., 1999; Pinhassi et al., 2004). More particularly, the genera *Alteromonas, Pseudoalteromonas* and *Vibrio* have been found to respond positively to dust additions (Laghdass et al., 2011; Guo et al., 2013). While these groups were present in our samples, we did not observe a significant increase in any of them with aerosols; however, *Oceanospirillaceae* did. Interestingly, *Oceanospirillaceae*, that have been observed to degrade polycyclic aromatic hydrocarbons (Dombrowski et al., 2016), were highly correlated with TOC in our experiments (OTU 5051, Figure 2B, 3B). This may mean that *Oceanospirillaceae* are able to degrade organic sources released by aerosols, which are mainly thought to be of recalcitrant nature (Sánchez-pérez et al., 2016).

We expected that AA would yield a larger stimulation of heterotrophic bacteria due to their higher content in nutrients, more particularly soluble P and organic compounds. We determined that AA released indeed more N (in the form of nitrate and ammonia) than SD, but similar amounts of TIP and TOC than SD (Table 2; see section “Aerosol Composition and Release of Nutrients into the Seawater”). TIP showed a higher leachability from the AA, though, showing a significantly higher concentration in these microcosms along incubation time, compared to the controls. In any case, we could not find a clear link between nutrients released by aerosols and the increase in the relative abundance of specific bacterial groups throughout the six experiments, due to the high variability observed between experiments. We did find, however, a clearer pattern regarding the stimulation of *Synechococcus* with SD, what we attribute to the lower competition with larger phytoplankton cells in this treatment. In contrast, nanoeukaryotes were found to increase significantly in the AA treatment (Marín et al., 2017a). Overall, these results show the importance of the initial conditions when assessing the effect of aerosols on microbial communities (Marañón et al., 2010; Teira et al., 2013; Marín et al., 2017b). Diverse experiments through time at a given location or covering a transect along the NW Mediterranean Sea during a certain season would help to further unveil the overall effect of aerosols on bacterial communities in the Mediterranean.

On the other hand, no significant changes in the abundance of any of the OTUs or marine groups were detected in Barcelona during winter or in the experiment carried out offshore. Bacterial communities were certainly very similar during winter (C and AA), and we did not find appreciable differences for other variables in this experiment either (Marín et al., 2017a,b). We attribute this lower disturbance of microbial planktonic communities to aerosols in winter to other available sources of nutrients during this time of year. However, we did expect a large disruption of the bacterial community in the oligotrophic offshore experiment that we did not observe. In fact, some differences between treatments become obvious from Supplementary Figure S3. For example, *Gammaproteobacteria* were more abundant in the AA samples, while *Cyanobacteria* were less favored with this treatment. In this experiment, although we did not find significant differences between treatments in terms of bacterial abundance, bacterial production and extracellular enzymatic activity were enhanced by AA (Marín et al., 2017b). Thus, some differences in the active fraction of the community may have been occurred, with *Gammaproteobacteria* probably contributing to these changes. Unfortunately, we did not subsample for transcriptomic analyses. Measuring 16S rRNA transcripts derived from cDNA, Guo et al. (2016) found that *Alphaproteobacteria* were the more responsive group to direct dust inputs, whereas *Gammaproteobacteria* seemed to benefit more from the increase in phytoplankton biomass. This might also explain why we did not find significant differences for *Alphaproteobacteria* as a group but just for specific OTUs, since we may have missed their metabolic response.

Overall, aerosol amendments induced changes in the proportion of different taxa in some experiments as compared to the unamended controls. Because of the realistically small amounts of aerosols added to the experiments, we did not find large differences in community structure that could overcome patterns in space or time. That is, changes in taxa owing to the different amendments of particular experiments were not as strong as trends for the different locations and seasons to overcome initial condition variability. This study therefore highlights the importance of interpreting aerosol impact studies on microbes at different initial conditions, especially if the aim is to gain a general understanding at the marine ecosystem level. As aerosol deposition trends increase and the impacted volume of surface ocean water decreases through global and climatic changes, in the future it is reasonable to expect larger impacts on bacterial community composition than shown here.

## Author Contributions

IM-B and FP conceived and designed the study and collected the environmental samples. IM-B collected the experimental data and analyzed output data with the assistance of JL and AA. IM-B wrote a first draft of the manuscript to which all authors contributed in subsequent revisions.

## Conflict of Interest Statement

The authors declare that the research was conducted in the absence of any commercial or financial relationships that could be construed as a potential conflict of interest.

## Footnotes

1 www.calima.ws

2 http://rtlgenomics.com/

## Abbreviations

AA: anthropogenic aerosols (this abbreviation is used for both the aerosols themselves and the microcosms amended with this type of aerosol)
BCN: Barcelona
BLA: Blanes
C: controls
OFF: offshore location
SD: Saharan dust (this abbreviation is used for both the Saharan aerosols themselves and the microcosms amended with this type of aerosol)
SP: spring
SP-BCN: experiment carried out in Barcelona during spring
SP-BLA: experiment carried out in Blanes during spring
SU: summer
SU-BCN: experiment carried out in Barcelona during summer
SU-BLA: experiment carried out in Blanes during summer
SU-OFF: experiment carried out offshore during summer
TIP: total inorganic phosphorous
WI: winter
WI-BCN: experiment carried out in Barcelona during winter
