# Supplementary material for: Atmospheric Deposition Impact on Bacterial Community Composition in the NW Mediterranean
Source: Front Microbiol. 2019 Apr 24;10:858. doi: 10.3389/fmicb.2019.00858 (PMC6491866; doi:10.3389/fmicb.2019.00858)
Supplement: Supplementary file 1 [file Data_Sheet_1.PDF]

Supplementary Material

Atmospheric deposition impact on bacterial community composition in the NW Mediterranean

Isabel Marín-Beltrán*, Jürg B Logue, Anders F Andersson, Francesc Peters

*** Correspondence:** isabel.marin.beltran@gmail.com

Supplementary Methods S1. In-detail description of aerosol collection, classification, and inoculum preparation.

Aerosol Collection

Aerosols – in the form of total suspended particles – were sampled in Barcelona at the Institut de Ciencies del Mar (41.39°N, 2.20°E) and in Blanes at the Centre d'Estudis Avançats (41.68°N, 2.80°E). Aerosol collection was carried out based on forecast presence/absence of Saharan dust (SD) intrusions to the Iberian Peninsula (provided by www.calima.ws; see further down): SD particles were collected on June, 7th (2013), August, 5th (2013), February, 18th (2014), and April, 1st – 3rd (2014) in Barcelona; and on February, 18th – 20th (2014) in Blanes (the same filter was employed for the two experiments carried out in Blanes). Anthropogenic aerosols (AA) were collected on April, 10th (2013), July, 4th (2013), August, 12th (2013), December, 17th (2013), January, 15th, 17th and 27st (2014), February, 17th (2014), and July, 22nd – 23th and 28th – 29th (2014) in Barcelona; and on January, 21st – 23rd (2014), February, 11th – 13rd (2014), March, 18th – 20th and 25th – 24th, May, 6th – 9th, and June, 2nd – 5th in Blanes. Once the gravimetric determination of the particulate matter collected on the filters was performed, filters were cut into two equal sections. Half of the filter was kept at 4 ºC and employed to characterize the chemical composition of the particles, and the other half was frozen at −20 ºC until used as inoculum in the amendment experiments.

Aerosol Classification

Classification of aerosols as SD or AA was based on (1) the predicted presence (SD) or absence (AA) of Saharan dust intrusions to the Iberian Peninsula and (2) subsequent verification of the collected aerosol filters via chemical analysis.

The former integrated four approaches: 1) interpretation of daily meteorological conditions and daily air mass trajectories calculated at noon for a given day and for five days ago (at 750, 1500, and 2500 m above sea level), using the model HYSPLIT (Hybrid Single-Particles Lagrangian Integrated Trajectories; http://ready.arl.noaa.gov/HYSPLIT.php); 2) maps of the Ozone Monitoring Instrument Aerosol Index (ftp://toms.gsfc.nasa.gov/pub/omi/images/aerosol/) as well as daily satellite images from NASA (http://oceancolor.gsfc.nasa.gov/SeaWiFS/HTML/dust.html); 3) results from simulations using the models SKIRON (University of Athens; Athens, Greece), DREAM (Barcelona Supercomputing Centre; Barcelona, Spain), and NAAPS (US Naval Research Laboratory at Monterrey; Monterrey, USA); and 4) application of the model HIRLAM-AEMET with regard to wind trajectories (http://www.aemet.es/es/eltiempo/prediccion/modelosnumericos/hirlam005). All

four approaches were integrated to provide a forecast of the presence or absence of SD intrusions to the Iberian Peninsula; hence, allowing sampling of aerosols at time points predicted to be of mineral (SD) or rather anthropogenic (AA) origin.

Chemical analysis of the collected filters was subsequently carried out to verify the aerosols' origin. The analyses were done following the methodologies found elsewhere (Moreno et al., 2006; Querol et al., 2001). For this purpose, a number of ratios among different chemical elements were calculated (P:Al, Fe:Al, Zn:Al, Pb:Al, Cd:Al, Si:Ca, Si:Fe, Al:Ca, Al:Fe, Ti:Ca and Ti:Fe). Threshold ratios of 0.012, 0.63, $1.01.10^{-3}$, $3.41.10^{-4}$, and $1.71.10^{-6}$ exist for P:Al, Fe:Al, Zn:Al, Pb:Al, and Cd:Al, respectively (Guieu et al., 2010, and references therein): observed ratios below and above these threshold values would classify the samples as of SD and AA, respectively. Moreover, high ratios of Si:Ca, Si:Fe, Al:Ca, Al:Fe, Ti:Ca and Ti:Fe would be attributed to SD (Nava et al., 2012). To further distinguish SD from AA, the enrichment factor (EF) was calculated for trace metals as described in Migon et al. (2001):

$$EF_M = \frac{(\frac{M}{Al})_{AE}}{(\frac{M}{Al})_{CR}} \quad \text{Eq.S1}$$

, where M is the concentration of the metal estimated in the aerosol (AE) or in the continental crust (CR). The concentrations of M and Al in CR are the ones reported by Wedepohl (1995). Ratios close to 1 are typical of crustal origin aerosols, while values above 10 normally belong to anthropogenically-derived aerosols (Migon et al., 2001).

Aerosol inoculum preparation

Particles were extracted from the filters into 250 ml of artificial seawater (37 g l^{-1}; NaCl EMSURE, Grade ACS, Merck; Darmstadt, Germany) by sonication for 20 min (7 kHz) in a Bandelin SONOREX Digital 10 P Ultrasonic bath (Sigma-Aldrich, Merck; Darmstadt, Germany). This solution was used to inoculate the microcosm stimulation experiments.

Supplementary Methods S2. In-detail description of PCR amplification and pyrosequencing, and subsequent sequence analyses.

PCR amplification and pyrosequencing. The bacterial hypervariable regions V1, V2, and V3 of the 16S rRNA gene were PCR amplified, using a forward and a reverse fusion primer 28F (5'-GAGTTTGATCNTGGCTCAG-3') and 519R (5'-GTNTTACNGCGGCKGCTG-3') (Handl et al., 2011), respectively. The primers were modified in advance according to the final configuration: AdaptorA-MID-28F and biotin-AdaptorB-519R (AdaptorA and B are 454 Life Sciences adaptor sequences; Branford, CT, USA). Multiplex identifiers (MID) were 8-10 nucleotides long and sample specific. Amplifications were performed in a 25-μl reaction volume made up of 1μl of template, 1μl of each primer (5 μM), and 22 μl of the Qiagen HotStar Taq master mix (Qiagen Inc; Valencia, CA, USA). Molecular grade water was used as negative control. Reactions were performed on ABI Veriti thermocyclers (Applied Biosytems; Carlsbad, CA, USA) according to the following thermal profile: 95 °C for 5 min, 35 cycles of 94 °C for 30 sec, 54 °C for 40 sec, and 72 °C for 1 min, and finalized by one cycle at 72 °C for 10 min. PCR amplicons were then pooled equimolarly and cleaned using the Agencourt AMPure XP purification kit (BeckmanCoulter Inc.; Brea, CA, USA). The final, pooled, amplicon was re-quantified and diluted accordingly, upon which it was used in emulsion PCR. Sequencing was performed on a 454 GS-FLX+ system (454 Life Sciences).

Sequence analyses. Pyrosequences were processed in QIIME (v 1.6) (Caporaso et al., 2011). After de-multiplexing and a first quality check, sequences were between 125 and 600 bp long, showed a quality score >25, contained no more than 2 mismatches in the primer sequences, and no homopolymers longer than 6 bp. To correct for reading mistakes, a DeNoiser algorithm (Reeder and Knight, 2009) was run only including the sequences that had passed the initial quality check. Denoised centroids and singletons were clustered into operational taxonomic units (OTUs) at a sequence identity level of 97% using UCLUST (Edgar, 2010). Prior to chimera detection and removal (ChimeraSlayer) (Haas et al., 2011), representative sequences were aligned according to the SILVA (Quast et al., 2013) alignment (release 108) using MOTHUR (v 1.33.3) (Schloss et al., 2009). The remaining sequences were again aligned and taxonomy was assigned according to the SILVA alignment (release 123). OTUs were assigned to a given group in case its representative sequence showed a BLAST hit to a reference sequence with an e-value $< 10^{-5}$. OTUs with an e-value above this threshold were classified as uncertain. Finally, pyrosequences that were either assigned as Archaea, Eukaryota, or uncertain, or contained fewer than 2 reads, were removed from the final dataset.

References

Caporaso, J. G., Kuczynski, J., Stombaugh, J., Bittinger, K., Bushman, F. D., Costello, E. K., et al. (2011). NIH Public Access. 7, 335–336. doi:10.1038/nmeth.f.303.QIIME.

Edgar, R. C. (2010). Search and clustering orders of magnitude faster than BLAST. *Bioinformatics* 26, 2460–2461. doi:10.1093/bioinformatics/btq461.

Guieu, C., Loÿe-Pilot, M. D., Benyahya, L., and Dufour, A. (2010). Spatial variability of atmospheric fluxes of metals (Al, Fe, Cd, Zn and Pb) and phosphorus over the whole Mediterranean from a one-year monitoring experiment: Biogeochemical implications. *Mar. Chem.* 120, 164–178. doi:10.1016/j.marchem.2009.02.004.

Haas, B. J., Gevers, D., Earl, A. M., Feldgarden, M., Ward, D. V, Giannoukos, G., et al. (2011). Chimeric 16S rRNA sequence formation and detection in Sanger and 454-pyrosequenced

Handl, S., Dowd, S. E., Garcia-Mazcorro, J. F., Steiner, J. M., and Suchodolski, J. S. (2011). Massive parallel 16S rRNA gene pyrosequencing reveals highly diverse fecal bacterial and fungal communities in healthy dogs and cats. *FEMS Microbiol. Ecol.* 76, 301–310. doi:10.1111/j.1574-6941.2011.01058.x.

Migon, C., Sandroni, V., and Béthoux, J. P. (2001). Atmospheric input of anthropogenic phosphorus to the northwest Mediterranean under oligotrophic conditions. *Mar. Environ. Res.* 52, 413–426. doi:10.1016/S0141-1136(01)00095-2.

Moreno, T., Querol, X., Castillo, S., Alastuey, A., Cuevas, E., Herrmann, L., et al. (2006). Geochemical variations in aeolian mineral particles from the Sahara-Sahel Dust Corridor. *Chemosphere* 65, 261–270. doi:10.1016/j.chemosphere.2006.02.052.

Nava, S., Becagli, S., Calzolai, G., Chiari, M., Lucarelli, F., Prati, P., et al. (2012). Saharan dust impact in central Italy: An overview on three years elemental data records. *Atmos. Environ.* 60, 444–452. doi:10.1016/j.atmosenv.2012.06.064.

Quast, C., Pruesse, E., Yilmaz, P., Gerken, J., Schweer, T., Yarza, P., et al. (2013). The SILVA ribosomal RNA gene database project: Improved data processing and web-based tools. *Nucleic Acids Res.* 41, 590–596. doi:10.1093/nar/gks1219.

Querol, X., Alastuey, A., Rodriguez, S., Plana, F., Ruiz, C. R., Cots, N., et al. (2001). PM10 and PM2.5 source apportionment in the Barcelona Metropolitan area, Catalonia, Spain. *Atmos. Environ.* 35, 6407–6419. doi:10.1016/S1352-2310(01)00361-2.

Reeder, J., and Knight, R. (2009). The "rare biosphere": a reality check. *Nat. Methods* 6, 636–637. doi:10.1038/nmeth0909-636.

Schloss, P. D., Westcott, S. L., Ryabin, T., Hall, J. R., Hartmann, M., Hollister, E. B., et al. (2009). Introducing mothur: Open-source, platform-independent, community-supported software for describing and comparing microbial communities. *Appl. Environ. Microbiol.* 75, 7537–7541. doi:10.1128/AEM.01541-09.

Wedepohl, K. H. (1995). The composition of the continental crust. *Geochim. Cosmochim. Acta* 59, 1217–1232.

Supplementary Figures

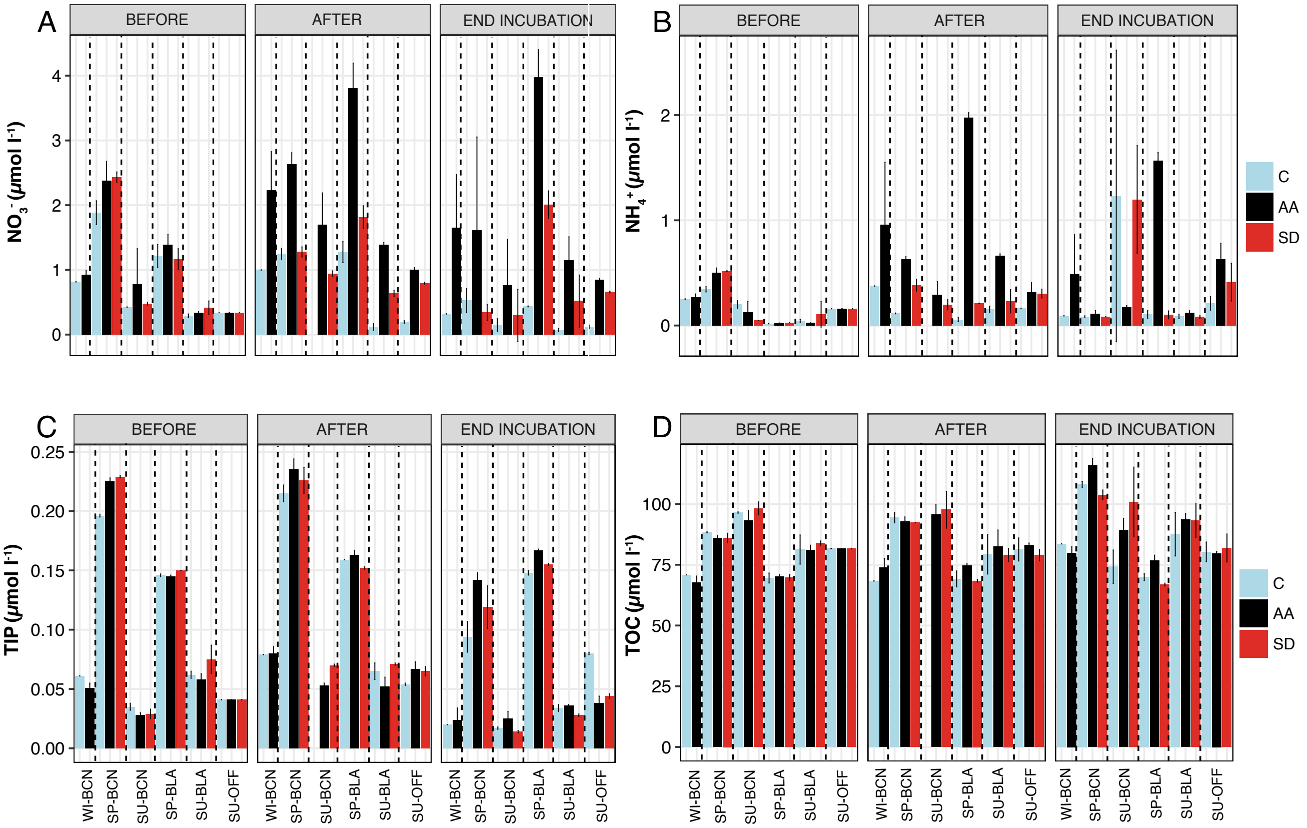

Figure S1. Average concentration of NO_3^- (A), NH_4^+ (B), total inorganic phosphorous (TIP) (C), and total organic carbon (TOC) (D) in each treatment (C, AA, SD). In each experiment, variables were measured before the aerosol addition ("BEFORE"), after the addition ("AFTER"), and at the end of the incubation time ("END INCUBATION"). Error bars show the standard deviation from two replicate containers (N = 2). Abbreviations: WI-BCN = winter experiment-Barcelona; SP-BCN = spring experiment-Barcelona; SU-BCN = summer experiment-Barcelona; SP-BLA = spring experiment-Blanes; SU-BLA = summer experiment-Blanes; SU-OFF = summer experiment-offshore; C = controls; AA = anthropogenic aerosols; SD = Saharan dust.

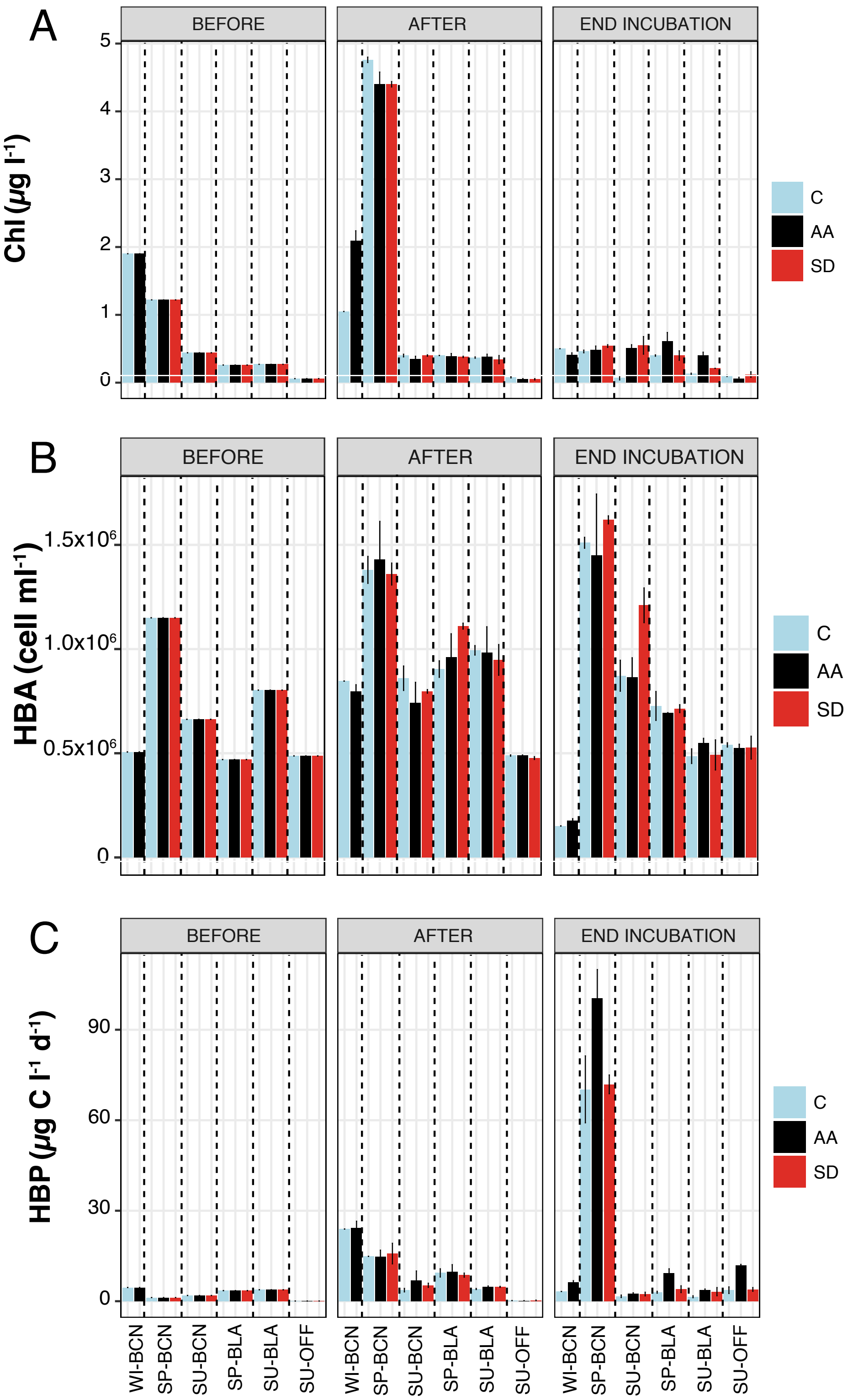

Figure S2. Average concentration of chlorophyll *a* (Chl) (A), heterotrophic bacterial abundance (HBA) (B) and production (HBP) (C) in each treatment (C, AA, SD). Legends and plot titles as in Figure S1.

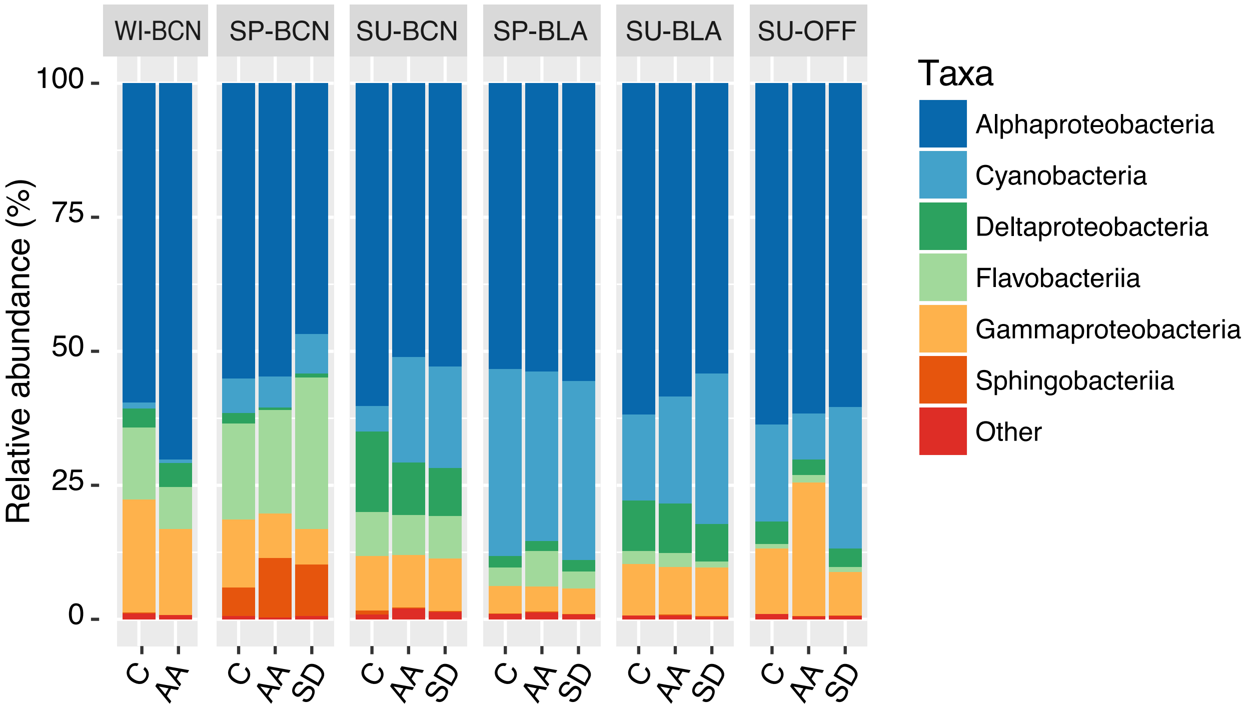

Figure S3. Relative abundance (%) of the most abundant marine groups (>1% of the total relative abundance) identified in the experimental samples, presented as the average from two duplicate microcosms ($N = 2$).

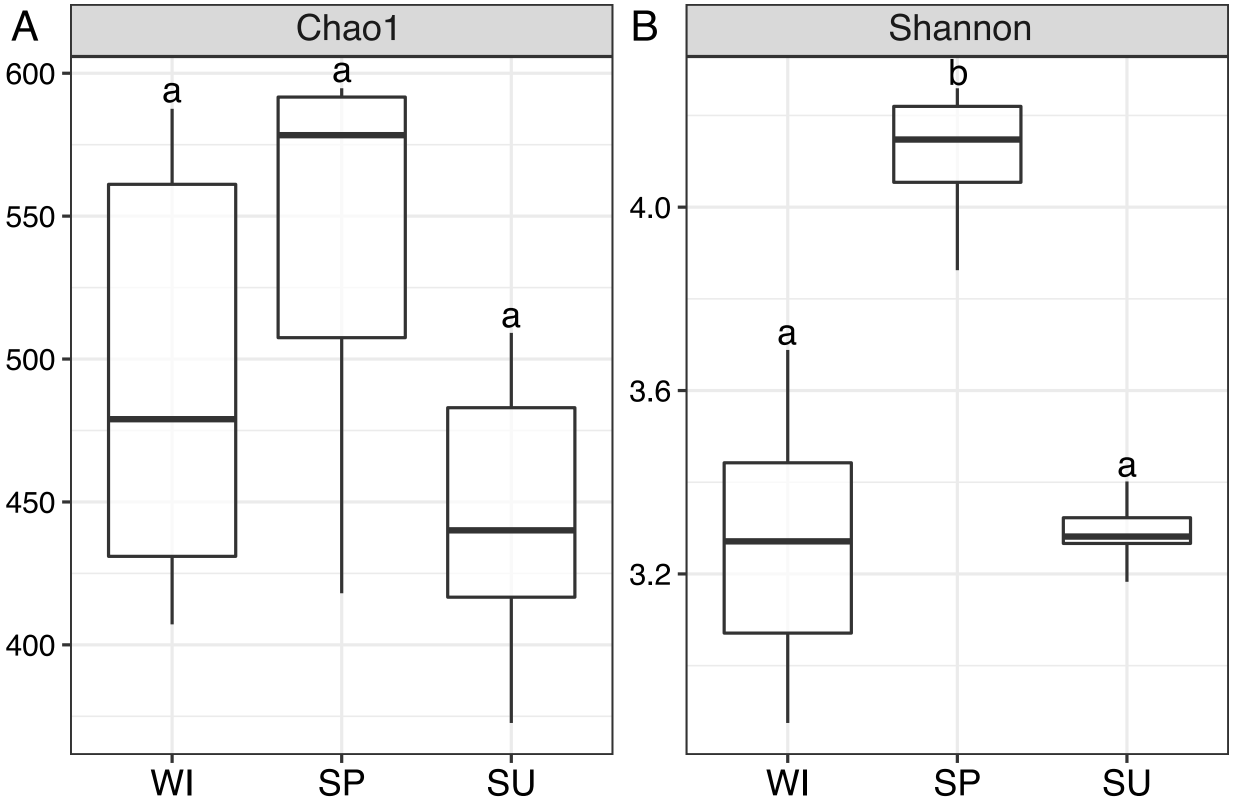

Figure S4. Box-plot representation of diversity indexes comparing the different seasons (WI, SP and SU) in Barcelona (*N* = 17). Diversity was measured by A) Chao 1 index, and B) Shannon index. The boxes indicate median and quartile values, while the whiskers indicate the range (minima and maxima). Different letters point to significant differences (PERMANOVA, $p < 0.05$) found between seasons.

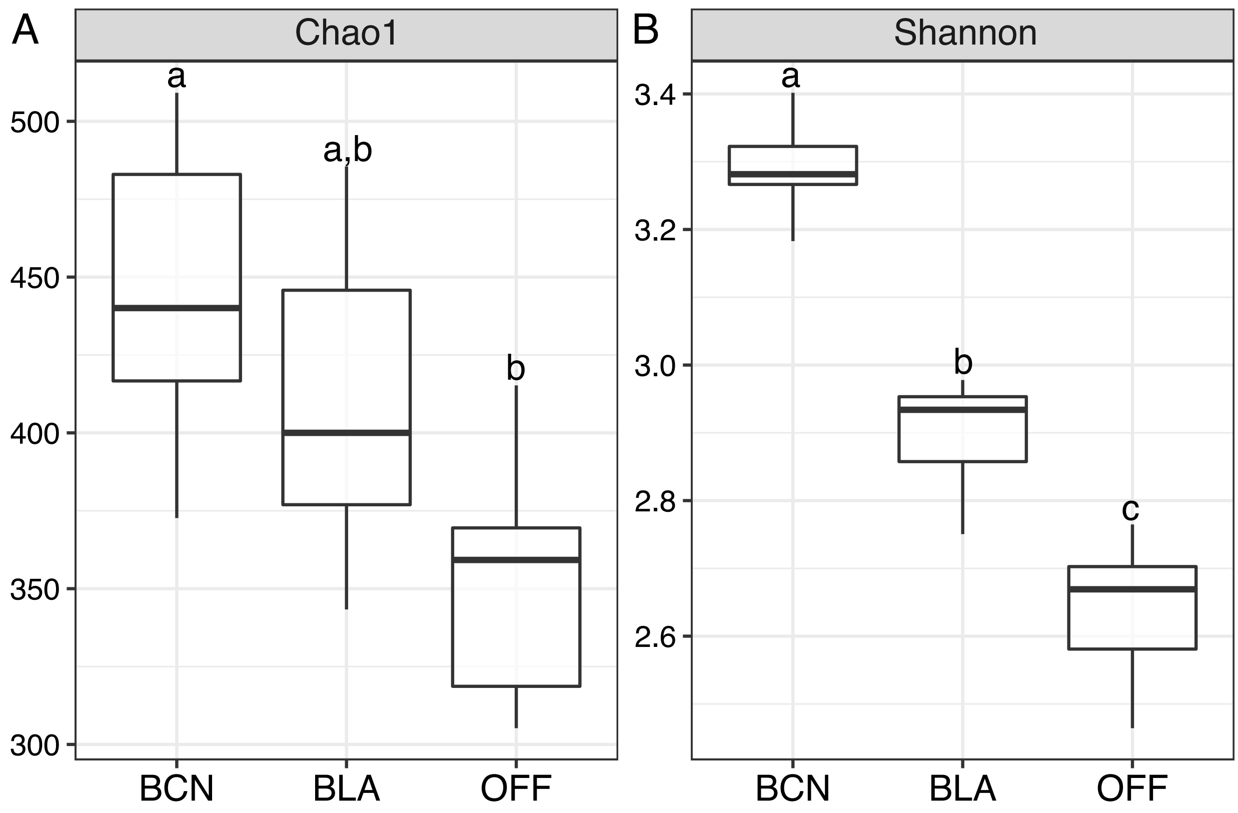

Figure S5. Box-plot representation of diversity indexes comparing the different locations (BCN, BLA, OFF) in summer ($N = 18$). Plot interpretation information as in Figure S4.

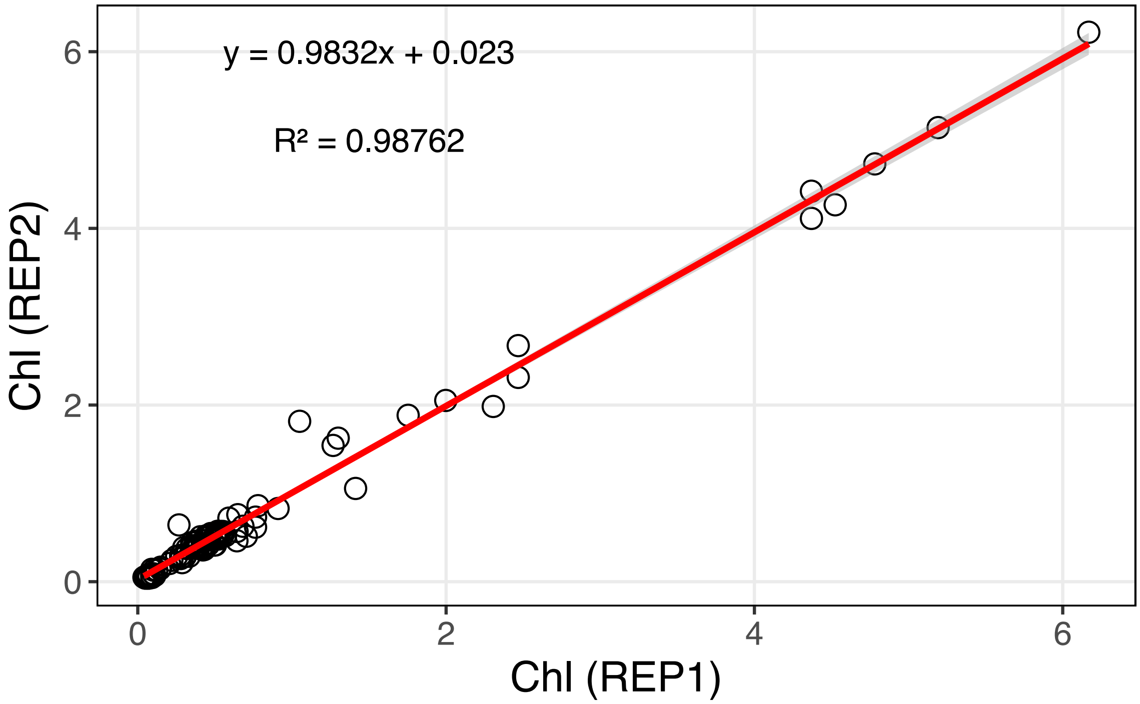

Figure S6. Linear regression between the two replicates for chlorophyll data measured in the 6 experiments, considering all the treatments and sampling dates (*N* = 192). The 95% confidence region is grey-shadowed around the regression line.

Supplementary Tables

Table S1. GenBank database accession number for the different samples.

Sample Name	Accession number
WI-BCN.C	SAMN05914900
WI-BCN.AAI	SAMN05914901
WI-BCN.AAII	SAMN05914902
WI-BCN.AAIII	SAMN05914903
WI-BCN.AAIV	SAMN05914904
SP-BCN.CI	SAMN05914922
SP-BCN.CII	SAMN05914923
SP-BCN.AAI	SAMN05914924
SP-BCN.AAII	SAMN05914925
SP-BCN.SDI	SAMN05914926
SP-BCN.SDII	SAMN05914927
SU-BCN.CI	SAMN05914888
SU-BCN.CII	SAMN05914889
SU-BCN.AAI	SAMN05914890
SU-BCN.AAII	SAMN05914891
SU-BCN.SDI	SAMN05914892
SU-BCN.SDII	SAMN05914893
SP-BLA.CI	SAMN05914905

SP-BLA.CII	SAMN05914906
SP-BLA.AAI	SAMN05914907
SP-BLA.AAII	SAMN05914908
SP-BLA.SDI	SAMN05914909
SP-BLA.SDII	SAMN05914910
SU-BLA.CI	SAMN05914928
SU-BLA.CII	SAMN05914929
SU-BLA.AAI	SAMN05914930
SU-BLA.AAII	SAMN05914931
SU-BLA.SDI	SAMN05914932
SU-BLA.SDII	SAMN05914933
SU-OFF.CI	SAMN05914936
SU-OFF.CII	SAMN05914937
SU-OFF.AAI	SAMN05914938
SU-OFF.AAII	SAMN05914939
SU-OFF.SDI	SAMN05914940
SU-OFF.SDII	SAMN05914941
